# *PLoS Computational Biology* Conference Postcards from PSB 2010

**DOI:** 10.1371/journal.pcbi.1000746

**Published:** 2010-04-29

**Authors:** Ruchira S. Datta, Matthew W. Lux, Philip E. Bourne

**Affiliations:** 1 Berkeley Phylogenomics Group, QB3 Institute, University of California Berkeley, Berkeley, California, United States of America; 2 Virginia Bioinformatics Institute, Virginia Tech, Blacksburg, Virginia, United States of America; 3 Department of Pharmacology, University of California San Diego, La Jolla, California, United States of America; 4 Skaggs School of Pharmacy and Pharmaceutical Sciences, University of California San Diego, La Jolla, California, United States of America

## Introducing PLoS Conference Postcards

### Philip E. Bourne, Editor-in-Chief, *PLoS Computational Biology*

Welcome to PLoS Conference Postcards. Postcards, as the name suggests, are designed to vividly recount to those not at a conference what happened at the event that was scientifically noteworthy and therefore make the reader wish they had attended. Postcards aim to be a departure from routine conference reports since they are written through the young and enthusiastic eyes of graduate students and postdoctoral fellows who intentionally focus on a small subset of what transpires at the meeting—from keynotes to research paper presentations to posters to working group discussions. In fact, Postcard writers may choose anything at the conference that is scientifically exciting. They are expected to accurately report on the aspect that excites them, but are free to provide additional commentary based on their own research into the topic or on the opinions of other participants, or after speaking with the speaker or poster presenter directly. Which Postcards are published is at the discretion of the conference organizers and PLoS editors, who carefully review all submissions. Successful submissions are published under the authors' names and indexed in PubMed.

The Pacific Symposium on Biocomputing (PSB), held on the Big Island of Hawaii from January 3–7, 2010, was our first call for Conference Postcards. What follows are two Postcards from that meeting, written by Ruchira Datta, a postdoctoral fellow in the laboratory of Kimmen Sjölander (University of California Berkeley), and Mathew Lux, a graduate student in the laboratory of Jean Peccoud (Virginia Tech). Interestingly, they independently chose to report on the presentation made by Edward Marcotte titled “Deaf Plants, Bleeding Yeast, and Other Surprising Disease Models”. The name alone suggests something noteworthy, but let's have the young people tell the story.

We will be experimenting with Postcards again at the forthcoming Intelligent Systems for Molecular Biology (ISMB) meeting to be held in Boston July 9–13, 2010, and expect to publish up to ten Postcards. So, either be ready to contribute a Postcard if you are a graduate student or postdoctoral fellow, or be ready to read an exciting account from the meeting if you will not be attending.

## Edward Marcotte on “Deaf Plants, Bleeding Yeast, and Other Surprising Disease Models” in the Dynamics of Biological Networks Session

### Reported by Ruchira S. Datta, University of California Berkeley

Those who chose to forego the delights of the Big Island to attend the final session of the conference were well rewarded with a stunning talk by Edward Marcotte on “Deaf Plants, Bleeding Yeast, and Other Surprising Disease Models.” Dr. Marcotte, a professor at the University of Texas at Austin, gave the invited presentation in the Dynamics of Biological Networks Session. Marcotte defines *phenologs* (http://www.phenologs.org) as significantly overlapping sets of orthologous genes, such that mutating any gene in a given set (from one organism) gives rise to the same phenotype in that organism. Surprisingly, sets of phenologs in yeast can predict genes leading to hemorrhaging, and sets of phenologs in plants can predict genes leading to congenital deafness, even though these disease states themselves have no analogs in these model organisms. Sets of phenologs continue to act as coherent subsystems over the course of evolution, albeit with dramatically different functions.

As the scientific community continues to produce an enormous flood of genetic and genomic information on population variation, a key emerging problem is how to correlate this information with phenotypic variation and disease. The aim is to construct a general model, preferably applicable to many diseases, that can be used to predict the phenotype resulting from a gene perturbation. The mutational phenotypes of model organisms have been measured systematically: many more genome-wide association studies have been performed in mouse, yeast, and worm than in human, and these should be exploited as far as possible.

**Figure 1:**
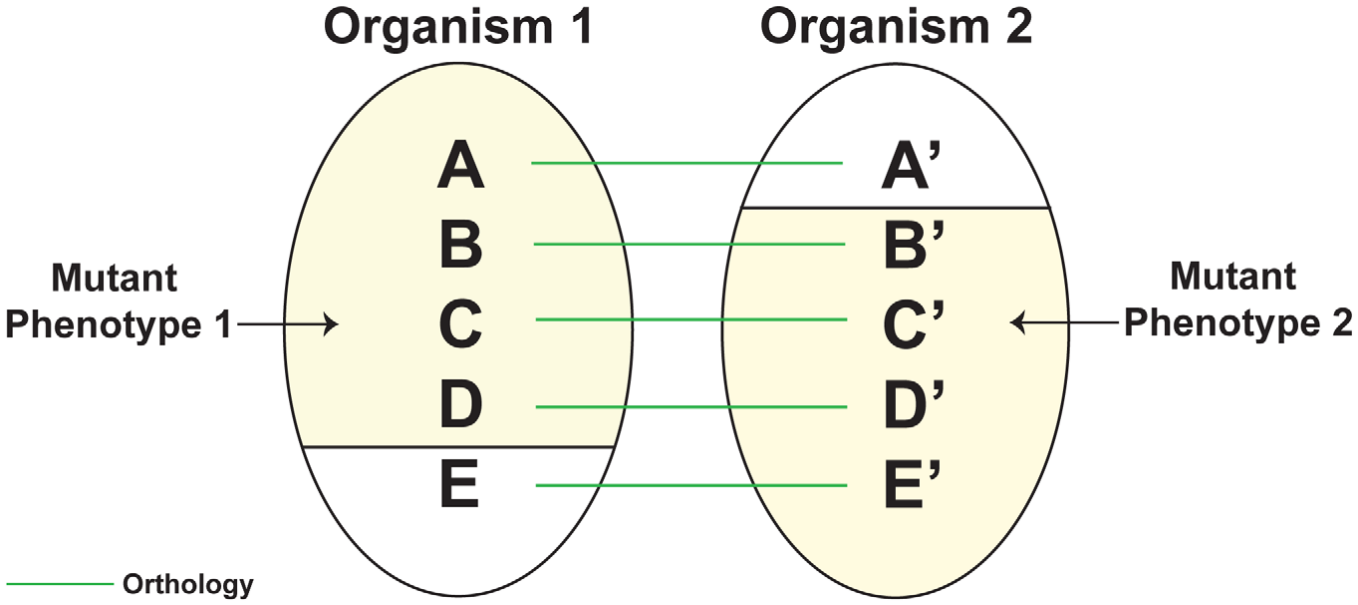
Phenologs.

Figure 1 illustrates the concept of phenologs. Here, genes A, B, C, D, and E in organism 1 are orthologous to genes A′, B′, C′, D′, and E′, respectively, in organism 2. Mutating A, B, C, or D leads to mutant phenotype 1 in organism 1. Mutating B′, C′, D′, or E′ leads to mutant phenotype 2 in organism 2. Thus, the set A, B, C, D, and E and the set A′, B′, C′, D′, and E′ are phenologs. Phenolog analysis predicts that mutating E would also lead to mutant phenotype 1, and mutating A′ would also lead to phenotype 2.

Phenolog analysis proceeds from gene–phenotype associations. Marcotte and his group mined databases and the literature to obtain these associations in human, mouse, worm, and yeast. To validate the phenolog analysis method, they conducted an all-pairs test between human disease gene sets and yeast phenotype gene sets. They corrected for multiple testing using permutation analysis. Assessing the results using cross-validation verified that the approach works to predict known disease genes. Weighting predictions from the top *k* phenologs worked better than predictions using single phenologs alone.

Some phenolog relationships are unsurprising. For example, mutating the orthologs in mouse of human genes that cause cataracts results in mouse cataracts. But Marcotte proceeded to provide examples demonstrating that this phenomenon extends much more widely than one might expect.

For instance, the statistical assessment found that whether a gene affected the sensitivity of yeast to lovastatin was a good predictor for whether it could cause angiogenesis defects in human. Of course, yeast have no blood vessels, so Marcotte wanted to validate this association experimentally. They checked whether the 62 genes predicted by phenolog analysis to relate to angiogenesis were expressed in the developing vasculature of frog embryos, and found that eight of them were (e.g., in the long vein or the developing heart). They also knocked down one of these genes, *SOX13*, in developing frog embryos. This caused severe angiogenesis defects, completely knocking out the veins and causing hemorrhaging in later stage embryos (thus, “bleeding yeast”). Marcotte and his group also verified angiogenesis defects in vitro in human umbilical vein endothelial cells.

Marcotte and colleagues found that even mutant phenotypes from such distantly related organisms as plants could be linked to human disease. For instance, the orthologs of genes causing cotyledon development defects lead to mental retardation, and the orthologs of genes causing defective response to red light lead to abnormal heart development. They validated one example in more detail.

The orthologs of genes causing negative gravitropism in plants lead to Waardenburg syndrome, a congenital human disease characterized by craniofacial, hearing, and pigmentation alterations (specifically, white forelocks). This syndrome accounts for 2%–5% cases of human deafness (hence, “deaf plants”). Waardenburg syndrome is a defect of neural crest cells, which migrate during embryonic development to give rise to the brain as well as to the arches of the ear, the craniofacial structure, and so forth. The phenolog correspondence with plant-negative gravitropism predicted a new neural crest cell effector, SEC231P, which does localize to neural crest cells. Marcotte and his group knocked this gene down in a frog embryo. In particular, they injected the antisense reagent precisely into a cell of the embryo such that only one side of the embryo received the treatment, and the other half served as the control. Indeed, visual inspection of the blue neural crest migrating cells shows their defectiveness on the treated side.

Marcotte and his group believe that phenolog analysis works by identifying evolutionarily conserved systems of proteins relevant to particular traits or diseases. Lending support to this hypothesis, they found that genes involved in phenolog relationships are more strongly interconnected in protein networks. Phenologs exhibit extremely distant homology (“deep homology”) of coherent molecular subsystems of proteins, reflecting the innate modularity of gene systems and identifying their adaptive reuse in different organisms.

Marcotte's presentation was well received by the attendees. The session concerned several different types of dynamics, such as spatial, temporal, tissue, or disease context, etc. Marcotte's work in particular illuminated the *evolutionary* dynamics of biological networks. This work is remarkable not just as a novel biocomputing method (whose utility will continue to be assessed in the course of time by other practitioners in the wider community), but especially as a striking biological discovery about how evolution works. Such a fundamental scientific advance exemplifies the best of what bioinformaticians hope to achieve.

## Edward Marcotte on “Deaf Plants, Bleeding Yeast, and Other Surprising Disease Models” in the Dynamics of Biological Networks Session

### Reported by Matthew W. Lux, Virginia Tech

The highlight of PSB for me was the invited talk by Dr. Edward Marcotte during the last session, Dynamics of Biological Networks. His work involved using computational methods to find good candidates for genes related to genetic disease, and then testing them experimentally. More specifically, Marcotte and his team looked across species for homology-based overlaps between pools of genes related to specific functions or phenotypes within their respective species. In other words, they looked for overlaps in genes related to function A in species 1 and genes related to function B in species 2. Importantly, the compared functions or phenotypes did not have to be in any way related; only the existence of overlapping homologous genes was necessary for two groups to overlap. Where overlaps were found, they looked more carefully at those genes that were connected to one species, but had no homolog associated to the function/phenotype grouping in the other species. Such genes were compared based on homology to genes of unknown function in the second species. Put another way, they looked for genes of unknown function in species 2 that had homology with genes that were of function A in species 1, but that were not in the intersection. Matches at this point were considered likely to have function associated with the intersecting phenotype found originally in the second species. While some of the intersecting functions/phenotypes were unsurprisingly very similar between species, others were notably quite disparate. Dr. Marcotte then proceeded to detail their investigation of a few of the most interesting of these candidates experimentally (i.e., those that came from intersections of very different function/phenotype groupings), showing results from knockout experiments in yeast and frog embryos.

This talk was the most memorable because it showcased so completely and concisely a successful example of one of the primary themes of the conference, namely using computational tools to leverage existing datasets to drive biological discovery. The work represents a paradigm for this type of research, as it meets each of the crucial elements of this theme: origins in computational analysis of existing data, experimental validation of computational predictions, and useful biological insights. For a variety of reasons, such research often lacks the strong balance of computation and experiment that was present in this work. In particular, the computationally driven biological discovery in this case ends in the uncovering of disease-related genes, a result of obvious implications and benefits and a real endorsement of the approach. Rarely does one presentation cover all of the goals of computationally driven biological discovery so adequately.

Another positive aspect of the talk was the high level of accessibility. As a graduate student with a relatively limited breadth of expertise, a problem I repeatedly had at PSB during sessions other than my own was getting mired in the technical details with which I was not familiar. By glossing over some of the finer details of the algorithms used to look for and analyze the overlaps in favor of focusing on the goals, motivations, and results of the computational work, Dr. Marcotte managed to elucidate the central important points of the research, even for audience members of varying interests. Likewise, the experimental setups and results were explained well enough to be convincing, but not in excessive detail that might otherwise bog down and cloud the primary message. I appreciated how the presentation was well balanced between high-level overview and sufficient detail, especially for a talk that covered both significant computational and experimental components.

The success of the talk was punctuated by the obvious impact on the audience. Lines at the microphones quickly formed, and there was not enough time to answer all of the questions. A further positive sign was that none of the questions were critical, only asking for Dr. Marcotte's opinion on future directions or for clarification of some of the skimmed-over points. The attendee next to me said that it was the best talk of the conference, and we discussed the various merits of the presentation. Though I did not get the chance to ask questions immediately after his talk, I spoke with Dr. Marcotte later in the day. Since he had only given a few examples on experimental validation, and knowing that the research was ongoing and unpublished, I wondered a) what percentage of their computationally uncovered “good candidate” genes had been tested experimentally, and b) what percentage of those that had been explored turned out to be successfully associated with the intersecting cross-species match. I learned that though they had only tested a few of the candidates in the lab, all of the predictions thus far had been validated. Despite the small sample size, such a success rate is encouraging. Not only do the results so far bode well for the wealth of potential discovery of disease-related genes through Dr. Marcotte's specific research, but they also provide a prime example of a main PSB theme demonstrated fully in practice. The research fortifies the idea that similar approaches of computationally driven biological discovery will increasingly provide far-reaching and useful results as we move towards the future.

